# Agreement between physicians and the InterVA-4 model in assigning causes of death: the role of recall period and characteristics specific to the deceased and the respondent

**DOI:** 10.1186/2049-3258-71-28

**Published:** 2013-11-06

**Authors:** Sebsibe Tadesse

**Affiliations:** 1 Institute of Public Health, the University of Gondar, Gondar, Ethiopia

**Keywords:** Agreement, InterVA model, Physician review, Recall period, Verbal autopsy

## Abstract

**Background:**

In the absence of routine death registration, the InterVA model is a new methodology being used as a physician alternative method to interpret verbal autopsy (VA) data in resource-poor settings. However, various studies indicate that there are significant discrepancies between the two approaches in assigning causes of deaths. This study evaluated the role of recall period and characteristics that were specific to the deceased and the respondent in affecting the level of agreement between the approaches.

**Methods:**

A population-based cross-sectional study was conducted from March to April, 2012. All adults aged ≥14 years and died between 01 January, 2010, and 15 February, 2012, were included in the study. Data were collected by using a pre-tested and modified WHO designed verbal autopsy questionnaire. The verbal autopsy interviews were reviewed by the InterVA-4 model and the physicians. Cohen’s kappa statistic with 95% CI was applied to compare the strength of the agreement between the model and the physician review.

**Results:**

A total of 408 VA interviews were successfully completed and reviewed by the InterVA model and the physicians. Both approaches showed an overall agreement in 294 (72.1%) of the cases [kappa = 0.48, 95% CI: 0.42 - 0.60]. The level of agreement between the approaches was *low* [kappa ≤0.40] when the deceased was female, 50 and above years old, single, illiterate, rural dweller, belonged to a family of 1–4 people living together, and died at home. This was also true when the recall period was ≤1 year, and the respondent was a relative other than parent/marital partner, lived with the deceased, and had medical information.

**Conclusion:**

This study identified important variables affecting the strength of agreement between the InterVA-4 model and the physician in assigning causes of death. The results are believed to significantly contribute to the process of identifying the actual underlying causes of deaths in the population, and may thus serve to promote informed health policy decisions in resource-poor settings.

## Background

Developing countries generally lack consistent, timely, and reliable information on levels of cause specific mortality fractions (CSMFs) in their populations [1,2]. Verbal autopsy (VA) is a useful tool in such settings to establish the probable cause of death (COD) by interviewing a close caregiver or anyone who can provide witness to the death event [3]. VA data are often reviewed by physicians in order to assign the probable COD. But in addition to being time and energy consuming, the method is likely to produce inconsistent results [4-16]. Different alternative methods to the physician review process for interpreting VA data have remained of limited use [17-19]. However, the use of the InterVA model to interpret VA data has just been explored to have the advantage of achieving the maximum spatial and temporal consistency in interpreting VA data. Moreover, it requires less time and labor resources, especially in comparison with the physician review method [20-22]. Also, it is freely available in the public domain, making it ideal for resource-constrained settings [23].

Various studies have been conducted to compare the performance of the InterVA model as a physician alternative method to interpret VA data [12,20,24,25]. However, the results still show some discrepancies in comparison to the physician review. Moreover, the role of recall period and characteristics specific to the deceased and the respondent in affecting the level of agreement between the two approaches have not been assessed. Therefore, this study is designed to evaluate the role of recall period and characteristics specific to the deceased and the respondent in affecting the level of agreement between the InterVA model and the physician. The study results are believed to significantly contribute to the process of identifying the actual underlying CODs in the population, and may thus serve to promote informed health policy decisions in resource-poor settings.

## Methods

A population-based cross-sectional study was carried out from March to April, 2012, in Dabat Health and Demographic Surveillance System site (HDSSs), hosted by the University of Gondar. The site is located in a district known as Dabat, northern Ethiopia, which has an estimated population of 46,165 living in 7 rural and 3 urban “kebeles” (the smallest administrative units in Ethiopia). The local communities largely depend on subsistence agriculture. Information on vital events, like birth, death, and migration are collected quarterly [26].

### Study population and data collection

All adults aged ≥14 years and died between 01 January, 2010, and 15 February, 2012, in the area were included in the study. This period was preferred in order to obtain an adequate number of deaths without a marked recall bias. It is believed that adult deaths were remembered very well.

Pre-tested and modified WHO and INDEPTH [27,28] designed VA questionnaire was used to collect the data. The VA questionnaire included open narrative, medical histories, and closed questions. The narrative section was used to record free explanations of the circumstances of deaths; the medical history sections were used to extract data from medical certificates, and the closed section dealt with specific signs, symptoms, and conditions leading to death. Three trained supervisors and nine data collectors who had rich experience in the job participated in the data collection processes. After obtaining informed written consents, the data collectors interviewed a close relative, a friend, or neighbors of the deceased person who witnessed the death.

The VA questionnaire was translated into “Amharic” (the local language) and back to English to maintain the consistency of the questions. The training of the data collectors and supervisors emphasized issues, such as the selection of eligible respondents, approaching grieving respondents, time of interviews, and compiling narrative responses (ensuring that duration, frequency, severity, and sequence of symptoms were mentioned). The principal investigator and the supervisors coordinated the interview process, made spot-checks, and reviewed the completed questionnaire on daily bases to ensure the completeness and consistency of the data collected .They also conducted random quality checks by re-interviewing about 10% of the respondents. The VA questionnaire was pre-tested to identify potential problem areas, unanticipated interpretations, and cultural objections to any of the questions on 25 respondents (near Dabat district) with characteristics similar to the study subjects. Based on the pre-test results, the questionnaire was adjusted contextually. Data entry was carried out by the principal investigator and another independent data clerk and was then compared to check for any variation in results.

### Interpretation of VA data

The InterVA-4 model and the physician reviewed the same basic data from the VA questionnaire independently. That is, both methods utilized information in the open narrative, medical history, and the closed-ended section to assign the probable COD.

### Physician interpretation

Two independent physicians reviewed each VA questionnaire independently to assign a single COD based on ICD-10. The ICD-10 list had unique codes for diseases, signs, symptoms, abnormal findings, complaints, social circumstances, and external causes of injury [22]. The physicians met subsequently to reach consensus on cases where there were differences of opinion. If no physician consensus was reached after discussion, the COD was regarded as indeterminate. The physicians were trained in procedures on assigning COD and given details of the study area and study population. However, they were not given any special briefings on the probabilistic model so as not to encroach on their professional freedom. In spite of that, however, their review process was closely monitored and that they be not direct beneficiaries of the research output was ensured.

### Interpretation of the InterVA model

The model relates a range of input indicators, such as age, sex, physical signs and symptoms, medical history, and the circumstances of death to likely CODs using the Bayesian probabilities [22]. The model results in up to three likely causes per case when possible; each associated with a quantified likelihood. To assign an estimate of the overall certainty for that patient, the model gives the average likelihood for a maximum of three CODs [23]. In this study, a high prevalence of malaria and HIV/AIDS were used as basic epidemiological parameters for the model as their prevalence varied from place to place. Data were entered into the already specified batchin.csv file format of InterVA version 4, and a readable text output log file format was chosen to assign the possible COD responsible for the death of each individual.

### Agreement between the InterVA model and the physician

The most probable CODs assigned by the model were considered to facilitate comparison with the single CODs which were assigned by the physician. In a case where there was more than one probable CODs provided by the InterVA-4 model, the second and the third, if any, CODs were considered to compare the agreement between the model and the physician reviews. Agreement between the two approaches was sought at chapter heading level of ICD-10. All CODs in both methods were re-categorized into 9 main groups for two reasons. The first reason was to have meaningfully comparable COD categories between both methods. Second, it was more important that the model and the physician arrive at a broad agreement in identifying COD groups with the greatest public health importance at population level, rather than individual level causes. The 9 main categories used in this study were the following: pulmonary tuberculosis, kidney diseases, liver diseases, diabetes, other infectious diseases, cardiovascular problems, maternity-related deaths, other non-communicable diseases, and injuries/accidents.

Then deaths were aggregated case-by-case to their respective COD categories in order to determine the CSMFs at the community level by using both the InterVA model and the physician review. Cohen’s kappa statistic (K) with 95% confidence interval (CI) was applied to compare the agreement between the InterVA model and the physician review. Complete agreement corresponds to a K of 1 and complete disagreement to a K of 0. The strength of agreement was rated as *low* for a K ≤ 0.40, *moderate* for a K between 0.41 and 0.60, *good* for a K between 0.61 and 0.80, and *very good* for a K > 0.80 [29].

### Ethical considerations

The study protocol was reviewed and approved by the Institutional Ethical Review Board of the University of Gondar. Then, informed written consent was obtained from the study participants who were close relatives, friends, or neighbors of the deceased after explaining the purpose and the procedures of the study. Confidentiality was guaranteed for information collected from each study participant. Study participants found sick at the time of data collection were referred to the nearest health institution for medical treatment. There was no remuneration for family.

Finally, for the purpose of completeness findings of the previous studies on the physician reviews of the VA data were included in this study [13,30]. The current and the previous studies were conducted in the same study area, data source, and study period.

## Results

A total of 408 VA interviews were successfully completed and reviewed by both the physicians and the InterVA model.

### Physician interpretation

Out of the 408 deaths, 329 (80.6%) were successfully assigned a single cause at the first attempt by two physicians. After holding consensus meetings, the physicians readily assigned a single COD to 61 (15%) more cases. Therefore, on the whole, physicians assigned a single COD to 390 (95.6%) cases. No consensus was reached on 18(4.4%) cases which were coded as “indeterminate” by the physicians.

### Interpretation of the InterVA model

The InterVA model assigned a single COD to 347 (85.1%) cases, two CODs to 40 (9.8%) cases, and three causes to 3(0.7%) cases. In 18 (4.4%) cases, the InterVA model assigned the COD as “indeterminate”.

### Agreement between the InterVA model and the physician

The level of agreement between the InterVA model and the physician in assigning CODs was evaluated in terms of recall period, and characteristics that were specific to the deceased and the respondent. A direct comparison of the CODs assigned by the physician to the CODs assigned by the InterVA model showed that there was an overall agreement in 294 (72.1%) cases [kappa = 0.48, 95% CI: 0.42 - 0.60]. There was a general similarity and just slight differences between the InterVA model and the physician in assigning CSMFs. Out of all deaths in this population, two major groups of causes, pulmonary tuberculosis and other infectious diseases, accounted for about half of the overall mortality, as determined by both approaches, (Table 1).

**Table 1:** Proportion of CSMFs by the physician and by the InterVA model in Dabat, Ethiopia from 01 January 2010–15 February 2012

	**CSMFs**	**By physician**	**By InterVA**
		**N = 408 (%)**	**N = 408 (%)**
1.	Pulmonary tuberculosis	94 (23.0)	120 (29.4)
2.	Kidney diseases	24 (5.9)	13 (3.2)
3.	Liver diseases	34 (8.3)	20 (4.9)
4	Diabetes	34 (8.3)	17 (4.2)
5.	Other infectious diseases	84 (20.6)	93 (22.8)
6.	Cardiovascular problems	41 (10.1)	31 (7.6)
7.	Maternity-related deaths	11 (2.7)	8 (2.0)
8.	Other non-communicable diseases	40 (9.8)	47 (11.5)
9.	Injuries/accidents	49 (12.0)	41 (10.0)
10.	Indeterminate	18 (4.4)	18 (4.4)

### The role of socio-demographic characteristics of the deceased

The level of agreement between the physician and the InterVA model in assigning CODs was *moderate* when the deceased was male, and *low* when the deceased was female. For the deceased who belonged to the age group of 15–49 years, and ≥50 years, the level of agreement was *moderate* and *low*, respectively. Regarding the marital status of the deceased, the level of agreement was *moderate* for married and *low* for single. The level of agreement was *low* when the deceased was illiterate and *moderate* when the deceased was literate. The level of agreement was *low* for rural residence and *moderate* for urban residence. Regarding the occupational status of the deceased, the level of agreement was *low* for farming occupation and government/private employment. The level of agreement was *low* when the family size of the deceased was 1–4 people and *moderate* when it was ≥5 members. For deaths that occurred at home, health facility and other places, the level of agreement was *low*, *moderate* and *moderate*, respectively (Table 2).

**Table 2:** Agreement between physicians and InterVA model CODs allocation by socio-demographic characteristics of deceased in Dabat, Ethiopia

**Variables number**	**Number**	**% agree**	**Kappa (95% CI)**
**Sex**			
Male	186	62.9	0.46 (0.4, 0.6)
Female	222	56.8	0.32 (0.2, 0.4)
**Age in years**			
15-49	127	67.7	0.59 (0.5, 0.7)
50-64	140	57.1	0.25 (0.2, 0.4)
≥65	141	54.6	0.37 (0.3, 0.5)
**Marital status**			
Single	83	58.5	0.36 (0.3, 0.5)
Married	325	63.9	0.56 (0.5, 0.7)
**Educational status**			
Illiterate	308	59.1	0.38 (0.3, 0.5)
Literate	100	61.0	0.43 (0.3, 0.5)
**Residence**			
Rural	306	59.8	0.36 (0.3, 0.5)
Urban	102	58.8	0.44 (0.3, 0.5)
**Occupational status**			
Farmer	298	58.8	0.42 (0.3, 0.5)
Gov’t/private employee	110	61.8	0.41 (0.3, 0.5)
**Family**			
1-4	282	58.9	0.37 (0.3, 0.5)
≥5	126	61.1	0.46 (0.4, 0.6)
**Death place**			
Home	336	55.7	0.36 (0.3, 0.5)
Health facility	32	62.5	0.50 (0.3, 0.7)
Other	40	90.0	0.54 (0.2, 0.8)

### The role of recall period

The level of agreement between the physician and the InterVA model in assigning CODs was *low* for a recall period of ≤1 year and *moderate* for a recall period of >1 year, (Table 3).

**Table 3:** Agreement between physicians and InterVA model CODs allocation by recall period and characteristics of respondent in Dabat, Ethiopia from 01 January 2010–15 February 2012

**Variables**	**Number**	**% agree**	**Kappa (95% CI)**
**Recall period**			
≤3 months	54	61.1	0.19 (0.0, 0.4)
4-6 months	68	57.3	0.32 (0.2, 0.4)
7-12 months	72	59.7	0.27 (0.2, 0.4)
13-18 months	116	62.9	0.53 (0.4, 0.6)
19-25 ½ months	98	56.1	0.44 (0.3, 0.5)
**Respondent relation**			
Parent/marital friend	180	61.7	0.42 (0.3, 0.5)
Other relative	172	55.2	0.33 (0.2, 0.4)
Unrelated	56	66.1	0.51 (0.3, 0.7)
**Respondent live with status**			
Yes	383	61.6	0.35 (0.3, 0.5)
No	283	58.7	0.41 (0.3, 0.5)

### The role of the characteristics of the respondent

When the relation of the respondent to the deceased was parent/marital partner, other relative and unrelated, the level of agreement between the physician and the InterVA model in assigning CODs was *moderate*, *low*, and *moderate*, respectively. The level of agreement was *low* when the respondent lived with the deceased and *good*, otherwise. It was *low* when respondents had medical information about the disease condition of the deceased and *moderate* when they didn’t, (Table 3).

## Discussion

In this study, the role of the socio-demographic characteristics of the deceased, recall period, and characteristics of respondents in influencing the level of agreement between the physician and the InterVA-4 model in assigning CSMFs at the population level was evaluated and found to be significant. A *moderate* level of agreement was found between the model and the physician in establishing all CODs [kappa = 0.48, 95% CI: 0.42 - 0.60]. Almost a similar finding was observed in a previous literature [31]. This indicated the temporal and spatial consistency of the model for establishing cause-specific mortalities.

The level of agreement between both approaches was *low* when the deceased was female as compared to when the deceased was male in this population. This could be explained by the low educational attainment combined with the poor health-seeking behavior of females [27,32-34] which might significantly influence correct symptom characterization of their illness conditions which in turn could possibly lead to wrong conclusions of the COD. Regarding the age of the deceased, a *low* level of agreement was observed for cases older than 50 and above as compared to younger ages. This could be justified by the simultaneous occurrence of multiple illness conditions with overlapping symptomatic nature as a result of age which might significantly influence the likelihood of COD assignment by both approaches. A *low* level of agreement was observed when the deceased was single as compared to married. This could be due to the fact that single people usually make infrequent and loose social interactions with the society. As a result, respondents could fail to correctly characterize the event responsible for the death when they are interviewed. Consequently, this might lead to confusion during COD assignment. There was a *low* level of agreement when the deceased was illiterate as compared to literate. The possible explanation for this could be that illiterate people seldom understand and explain their illness conditions correctly to their relatives which might significantly contribute to the *low* level of agreement between the physician and the InterVA model in assigning CSMFs at the population level. For rural residents, the level of agreement between the physician and the InterVA model was *low* compared to urban dwellers. This could be due to the fact that rural people in developing countries rarely seek appropriate modern medical services to correctly characterize their illness conditions although they suffer from multiple illness conditions with overlapping symptoms which might be responsible for the *low* level of agreement between the physician and the InterVA model. The reason for the *low* level of agreement between the physician and the InterVA model when family size of the deceased was 1–4 people might be that the low frequency of social contacts made by the deceased during their illness with the few family members who are already engaged in busy daily activities could directly impair their ability to correctly characterize the illness condition of the deceased when they are interviewed. Consequently, the physician and the InterVA model could reach an agreement on only few CODs. A l*ow* level of agreement was observed when the place of death was the home as compared to death at a health facility and other places. This could be explained by the fact that the majority of the people in the study area were illiterate who used more local terms to characterize the death event which could lead the two approaches to reach different conclusions.

Currently, a wide range of recall periods from the time of death to the interview is used in VA. Some perform interviews as soon as possible after death while others visit the household of the deceased after a minimum of four weeks to allow attendants an adequate mourning period. The maximum recall period varied from six months to an indefinite period. The effects of recall may differ depending on the context, characteristics and demographics of the deceased [35]. Validation studies confirmed that a recall period ranging from 1 month to 2 years is generally thought to be acceptable [36,37]. This study supports this finding on the ground that the level of agreement between the physician and the InterVA model in assigning CODs increased as the recall period got longer and longer. This could be so because as the time between the interview and death increased, there would be a decrease in respondent related bias enabling the respondent to characterize the death event freely. This could consequently improve the level of agreement between the two approaches in assigning the COD.

In this study, a *low* level of agreement between the physician and the InterVA model in assigning CODs was observed when the relation of the respondent with the deceased was other relative (son, daughter, brother, sister, uncle, ant) as opposed to parent/marital partner, and unrelated. The reason for this could be that other relatives would not spend most of their time with the deceased during their illness and try to characterize the illness condition wrongly when they are asked to explain the death event. This would consequently lower the level of agreement between the approaches in assigning the COD. Unlike this, parents/marital partners are more likely to spend most of their time with the deceased during their illness and as a result could explain the death event more accurately which may lead to an improved level of agreement in assigning the COD. Unrelated respondents rarely introduce respondent related bias when they are asked to explain the death event. This could contribute to the increased level of agreement between the approaches in assigning the COD. The reason for the *low* increase in the level of agreement when respondents lived with the deceased might be due to the influence of respondents’ traditional understanding and stereotyped way of characterizing the illness condition responsible for the death event. The level of agreement between the physician and the InterVA model in assigning CODs was observed to be *low* when the respondents had medical information about the disease condition responsible for the death event. The possible explanation for this could be that respondents who had medical information stick only to naming the specific-cause of death, ignoring the other indicators responsible for the death event when they were interviewed. This could affect the validity of the physician and the InteVA model differently.

The possible limitation of this study could be that the influence of the respondent’s age, sex, marital, occupational, and educational status on the level of agreement between the two approaches was not evaluated. Contextually and demographically sensitive VA studies should be conducted to address these gaps.

## Conclusion

In this study, a *low* level of agreement was observed between the InterVA-4 model and the physician in assigning CODs when the deceased was female, 50 and above years old, single, illiterate, rural dweller, belonged to a family of 1–4 people living together, and died at home. This was also true when the recall period was ≤1 year, and the respondent was a relative other than parent/marital partner, lived with the deceased, and had medical information. Therefore, in addition to providing adequate training to data collectors on how to select interviewees and elicit the right indicators of the COD responsible for a particular death, VA researchers should choose the appropriate recall period in order to generate high quality data to be used by the InterVA model. These techniques significantly contribute to the process of identifying the actual underlying CODs in the population, and may thus serve to promote informed health policy decisions in resource-poor settings.

## Competing interests

The author declares that he has no competing interest.

## Authors’ contributions

ST was entirely responsible for this paper.
